# Delivery of heliox with a semi-closed circuit during spontaneous breathing: a comparative economic theoretical study

**DOI:** 10.1186/s12890-015-0060-9

**Published:** 2015-06-10

**Authors:** Ivana Jurickova, Karel Roubík, Martin Muller

**Affiliations:** Department of Biomedical Technology, Faculty of Biomedical Engineering, Czech Technical University in Prague, Prague, Czech Republic; Department of Anesthesiology, Resuscitation and Intensive Care Medicine, First Faculty of Medicine Charles University in Prague and the Military University Hospital in Prague, Prague, Czech Republic

**Keywords:** Heliox, Helium, Airway obstruction, Ventilation, Semi-closed circuit

## Abstract

**Background:**

Heliox is a mixture of oxygen and helium which reduces airway resistance in patients with airway obstruction. In clinical practice, patients breathing spontaneously receive heliox via an open circuit. Recently, a semi-closed circuit for heliox administration has been proposed which minimizes consumption of heliox and therefore cost of the heliox therapy; although, the semi-closed circuit is associated with additional costs. The aim of the study is to conduct an economical analysis comparing total cost of heliox therapy using an open versus a semi-closed circuit in spontaneously breathing patients with airway obstruction.

**Methods:**

Four different systems for heliox administration were analyzed: an open circuit and three alternatives of a semi-closed circuit involving a custom made semi-closed circuit and two standard anesthesia machines. Total costs of heliox therapy were calculated for all the systems. For calculation of gas consumption, the clinical procedures limiting continuous heliox therapy including the aerosol therapy, personal hygiene and nutrition were taken into account. A sensitivity analysis was conducted for main input variables that may influence the results of the study.

**Results:**

Price of gases consumed by a semi-closed system represents less than 20 % of price of gases when a standard open circuit is used. This represents a saving of approximately 540 EUR per patient. The initial cost of the custom-made semi-closed circuit recuperates after treatment of 18 patients. The corresponding number of patients is 32 when a low-cost anesthesia machine is initially acquired and rises to 69 when a highly advanced anesthesia machine is considered.

**Conclusions:**

Heliox therapy in spontaneously breathing patients using a semi-closed circuit becomes more cost-effective compared to the open circuit, currently used in clinical practice, when applied in a sufficient number of cases. The impact of finding a cheaper way of heliox administration on the clinical practice needs to be ascertained.

## Background

Severe airway obstruction accompanies many diseases of various etiologies. Nosological units characterized by airway obstruction include bronchial asthma, chronic obstructive pulmonary disease (COPD), epiglottitis, laryngitis, tracheitis, bronchiolitis, tracheal or bronchial stenosis. Severe airway obstruction induces increased work of breathing, decreased alveolar ventilation and development of respiratory insufficiency leading to respiratory failure, often treated by mechanical ventilation. Invasive mechanical ventilation is associated with pulmonary and extrapulmonary complications and complications caused by the upper airway management [1].

Inhalation of heliox (a mixture of oxygen and helium) instead of air significantly reduces airway resistance due to the very low density of helium compared with nitrogen as the main component of air [2]. Therefore, heliox reduces work of breathing and improves gas exchange in a variety of respiratory conditions [3].

Despite lack of large, adequately powered, randomized controlled clinical studies, heliox is believed to postpone intubation and reduce intubation rate [4]. This represents the main benefit of heliox compared to the standard therapy using invasive ventilation which necessitates intubation.

In spontaneously breathing patients, heliox is administered using an open circuit via a facemask connected to a reservoir bag with a sufficient inflow to keep it inflated [5]. The consumption of heliox is very high in the open circuit, typically more than 18 L/min, in order to prevent rebreathing of the expired gas. Heliox therapy using the open circuit is associated with high costs. Heliox is relatively expensive compared to oxygen, but is much less expensive than mechanical ventilation [6]. Thus, the high cost of heliox represents a disadvantage of heliox therapy and contributes to limitation of its routine clinical use.

In order to reduce costs of heliox therapy, a semi-closed circuit for heliox administration has been developed [7]. This way of heliox administration in patients with airway obstruction allows a decrease in heliox consumption to even less than 1 L/min. However, one should consider higher initial cost of the equipment, additional nursing time and other requirements while using a semi-closed circuit.

The aim of the study is to investigate whether the administration of heliox using a semi-closed circuit represents a cost effective alternative to a standard way of heliox administration in spontaneously breathing patients with severe airway obstruction.

## Methods

**Fig. 1:**
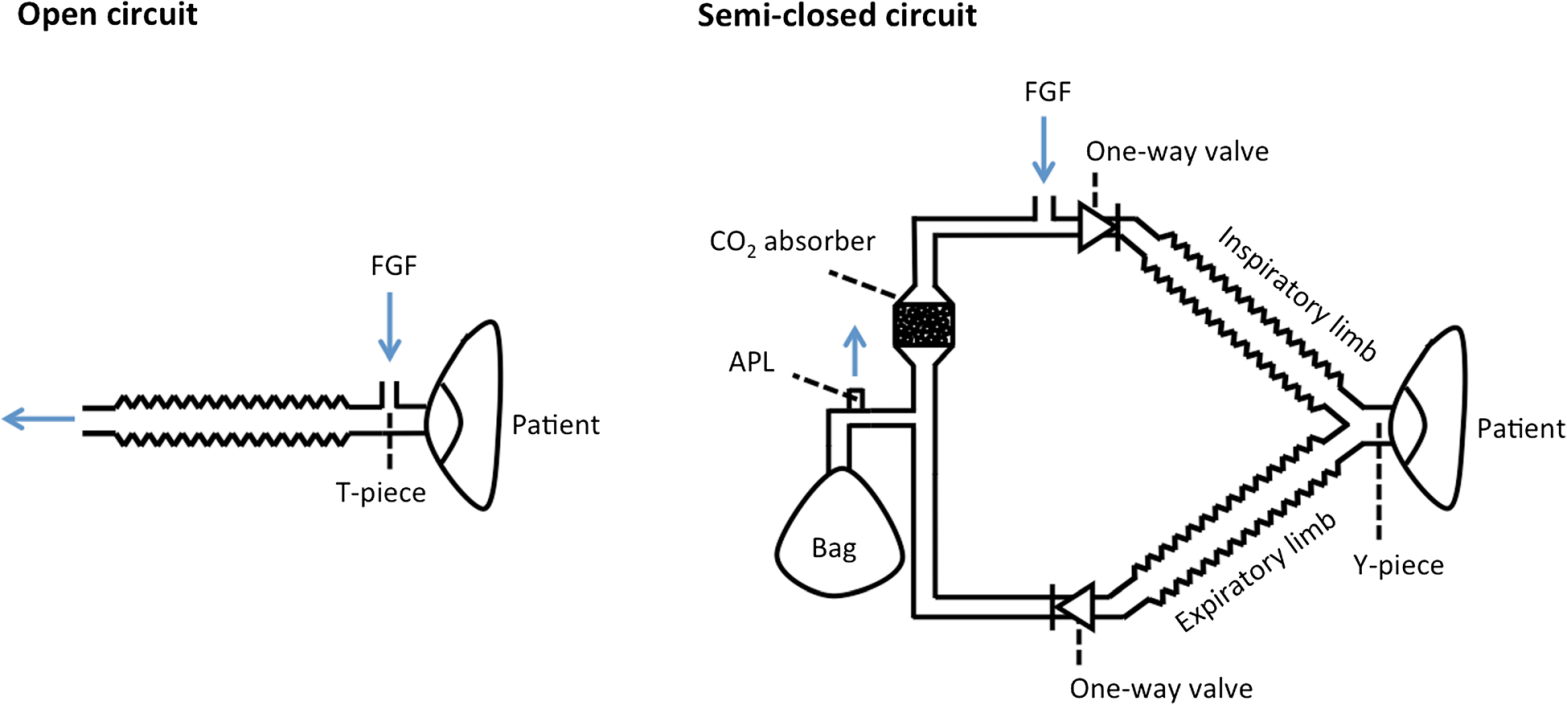
The schs. of an open circuit and a semi-closed circuit. FGF—Fresh Gas Flow, APL— Adjustable Pressure Limiting valve

Cost analysis of two different ways of heliox administration using four actual systems was conducted in this theoretical study. The first way is represented by an open circuit with a face mask which is the standard way of heliox administration in spontaneously breathing patients with severe airway obstruction. The second way of heliox administration is analogical to low-flow anesthesia using a semi-closed circuit where the fresh gas flow is significantly reduced. Currently, a commercially available semi-closed system for heliox administration is not available. Therefore, we considered two possibilities of practical realization of this system. The first choice is a custom made system comprising standard components of anesthesia machines and breathing circuits. Such a system is described in details in a recently published study [7]. The comparison of the semi-closed circuit with an open circuit is illustrated in Fig. 1.

The second alternative is to use a standard anesthesia machine. In order to compare all the possibilities, four different systems for heliox administration were compared: a standard open circuit with a mask for non-invasive ventilation, a custom made semi-closed circuit and two standard anesthesia machines differing in their acquisition costs: a low-cost anesthesia machine and an advanced anesthesia machine.

The initial and service costs of these four systems differ significantly. The common part of the open circuit consists of a pressure reducing valve with a Thorpe or gauge flowmeter and a piece of hose only. In contrast, the initial and service costs of the custom made circuit and an anesthesia machine are much higher. The initial and service costs of all the systems used for the analysis are presented in Table 1. The price of the custom made semi-closed circuit consists of the prices of all the components used in a prototype [7] and the price of its assembly by a commercial company. As the prices of standard anesthesia machines differ widely, we analyzed both the low-cost and the advanced anesthesia machines.

**Table 1:** Data used for the cost analysis

	Open circuit	Semi-closed circuit		
		Custom made circuit	Low-cost anesthesia machine	Advanced anesthesia machine
Initial costs and service (EUR)				
Price of device	311.83	7 120.02	14 858.97	31 183.01
Training	66.26	194.89	194.89	194.89
Service for 10 years	0.00	2 330.87	2 330.87	5 840.68
Total initial and service costs (EUR), *C*_TIS_	378.09	9 645.78	17 384.74	37 218.58
Costs per patient (EUR)				
Cost of consumables per a patient	9.90	13.56	13.56	13.56
Cost of sterilization per a patient	0.00	0.78	0.78	0.78
Cost of gases per a patient	673.55	132.25	132.25	132.25
Total costs per patient (EUR), *C*_TPP_	683.45	146.59	146.59	146.59

Costs of training for the open circuit and both the anesthesia machines are fixed by the manufacturers. The cost of training for the custom made semi-closed circuit was estimated as an average value of the training costs for both the anesthesia machines.

The cost of service for the open circuit and both the anesthesia machines represent the real prices of Safety technical inspections of the corresponding devices calculated for the whole lifetime which is considered to be 10 years. The cost of service for the custom-made circuit is equal to the service cost of the low-cost anesthesia machine as the complexity of these two systems is comparable.

Cost of consumables calculated per patient is also presented in Table 1. For an open circuit, the price of consumables per patient comprises prices of a T-piece, a piece of hose, and a high concentration face mask (Hi-Ox^80^, Viasys Healthcare, Yorba Linda, CA, USA). For a semi-closed circuit, the costs per patient comprise prices of a Y-piece, soda lime and an NIV face mask (NIV 7500 series V2 mask, Hans Rudolph, Shawnee, KS, USA) divided by the maximum sterilization cycles recommended by its manufacturer plus one additional time.

Cost of sterilization per patient related to a semi-closed circuit encompasses sterilization costs of an oro-nasal mask for non-invasive ventilation (NIV 7500) and components of the whole patient breathing system comprising of CO_2_ absorber, one-way valves, breathing bag and an expiratory valve.

**Fig. 2:**
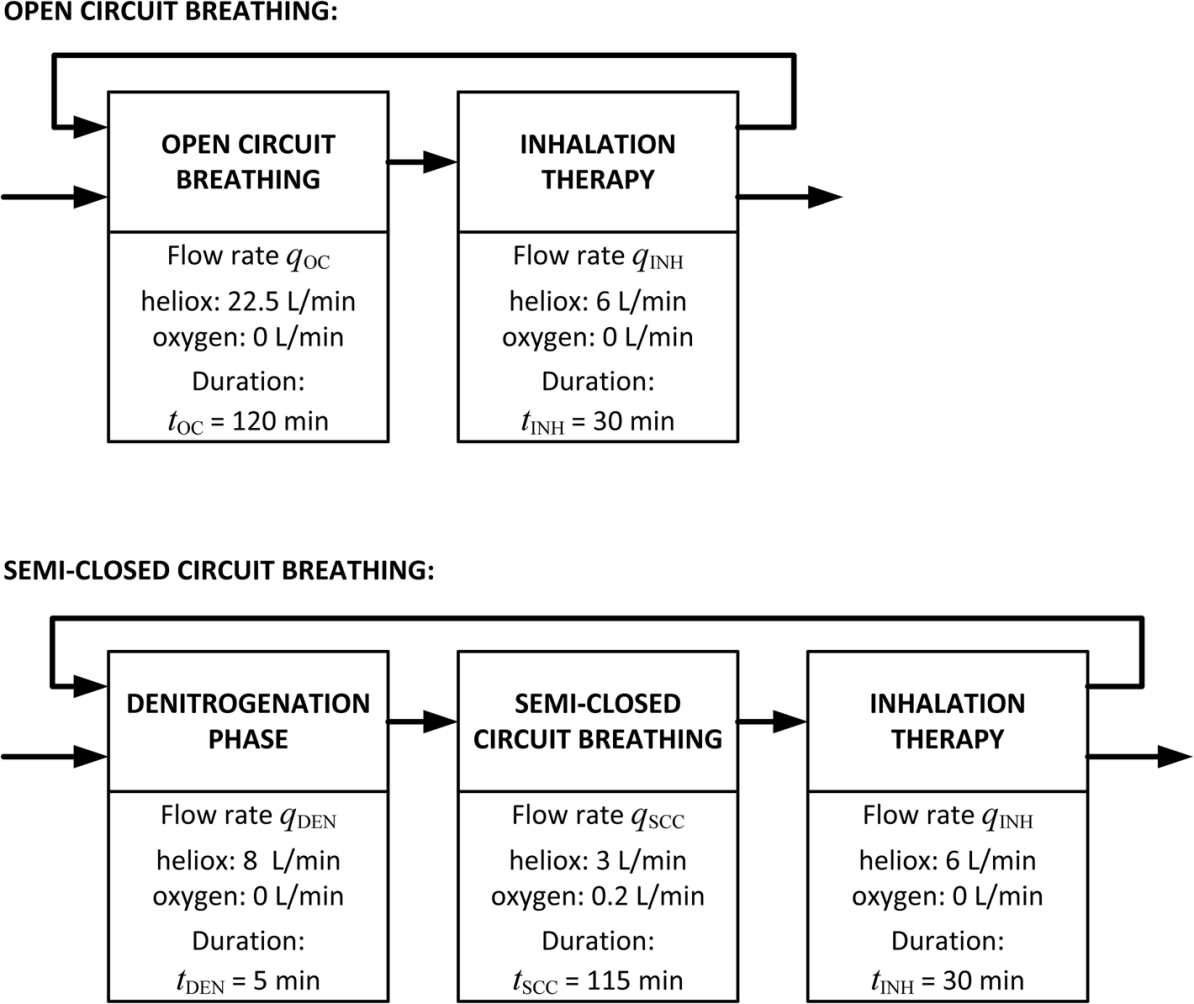
The basic treatment cycles during heliox administration using an open circuit (top) and any realization of a semi-closed circuit (bottom)

For calculation of the cost of gases (heliox and oxygen) consumed per patient, 25 h was considered as the average length of heliox therapy in spontaneously breathing patients. A detailed analysis of heliox and oxygen consumption during the therapy was conducted for each evaluated system. Heliox cannot be administered continuously because of the intermittent need for aerosol therapy, personal hygiene and nutrition. Furthermore, due to a low fresh gas flow into the semi-closed circuit, a phase of denitrogenation should be inserted in the beginning. This phase of denitrogenation uses a higher fresh gas flow (approx. 8 L/min) and it is equivalent to denitrogenation phase during a low-flow or minimum-flow anesthesia as depicted in Fig. 2. The application of heliox in a single patient can be therefore described as *N*_C_ repeated cycles consisting of two (for the open circuit) or three (for any realization of the semi-closed circuits) phases as depicted in Fig. 2.

As the total length of the heliox therapy including the periods of aerosol inhalation therapy is considered to be 25 h and each cycle lasts 150 min (2.5 h), the number of cycles per patient is *N*_C_ = 25/2.5 = 10. The total volume of gases (heliox or oxygen) consumed by a patient can be calculated as follows:

1 $$ {V}_{\mathrm{OC}}=\left({q}_{\mathrm{OC}}\cdot {t}_{\mathrm{OC}}+{q}_{\mathrm{INH}}\cdot {t}_{\mathrm{INH}}\right)\cdot {N}_{\mathrm{C}}, $$

2 $$ {V}_{\mathrm{SCC}}=\left({q}_{\mathrm{DEN}}\cdot {t}_{\mathrm{DEN}}+{q}_{\mathrm{SCC}}\cdot {t}_{\mathrm{SCC}}+{q}_{\mathrm{INH}}\cdot {t}_{\mathrm{INH}}\right)\cdot {N}_{\mathrm{C}}, $$

where *V*_OC_ (L) stands for the total volume of gas consumed by a patient during the whole therapy using an open circuit, *V*_SCC_ (L) stands for the total volume of gas consumed by a patient during the whole therapy using a semi-closed circuit, *q*_OC_ (L/min) is gas flow rate during the high flow therapy using an open circuit, *q*_INH_ (L/min) is gas flow rate during the inhalation, *q*_DEN_ (L/min) is gas flow rate during the denitrogenation, *q*_SCC_ (L/min) is gas flow rate during the low flow therapy, *t*_OC_ (min) is duration of high flow therapy, *t*_INH_ (min) is duration of inhalation, *t*_DEN_ (min) is duration of denitrogenation, and *t*_SCC_ (min) is duration of low flow therapy.

Costs of gases per patient presented in Table 1 were calculated using the average gas consumption computed using Equations (1) and (2) and using prices of gases listed in Table 2. The prices of gases contain the delivery costs and are calculated assuming that the healthcare facility owns the gas cylinders and therefore they do not pay a rental fee.

**Table 2:** Price of gases

Gas	Cylinder	Price per cylinder (EUR)	Volume at A.T.P.D. (L)	Price per liter at A.T.P.D. (EUR)
Heliox	50 L at 200 bar	233.87	10 000	0.0234
Oxygen	50 L at 200 bar	46.77	10 000	0.0047

*Abbreviation*: *A.T.P.D.* Ambient Temperature and Pressure, Dry

The main parameter assessed in this study was a total cost of heliox therapy per patient in euros per patient (*C*_HTPP_) covering gases, initial and other costs and respecting a number of patients that would undergo the heliox therapy calculated as follows:

3 $$ {C}_{\mathrm{HTPP}}=\frac{C_{\mathrm{TIS}}}{N_{\mathrm{P}}}+{C}_{\mathrm{TPP}}, $$

where *C*_HTPP_ is the resultant cost of heliox therapy per patient, *C*_TIS_ stands for the initial and service costs, *C*_TPP_ for total costs per patient and *N*_P_ stands for a total number of expected therapy cycles provided with the assessed system, i.e., a number of patients when no repeated applications are assumed. Values of *C*_TIS_ and *C*_TPP_ were taken from Table 1. Dependence of the total cost of heliox therapy per patient, *C*_HTPP_, was presented in a form of graph for all fours assessed heliox administration systems in order to allow for comparison.

Only two parameters may change and affect *C*_HTPP_ significantly and were studied during the consequent sensitivity analysis. The first variable studied was the price of heliox. *C*_HTPP_ were computed for heliox prices if they were increased or decreased by 25 %. The second variable studied was the patient’s minute ventilation. The average minute ventilation of 7.5 L/min which corresponds to healthy patients was increased to 10 L/min, 12.5 L/min and 15 L/min during the sensitivity analysis.

## Results

Price of gases consumed by a patient is significantly higher when a standard way of heliox administration using an open circuit is used compared to a semi-closed circuit. Price of gases consumed by a semi-closed system represents less than 20 % of price of gases when a standard open circuit is used which represents a saving of approximately 540 EUR per patient.

**Fig. 3:**
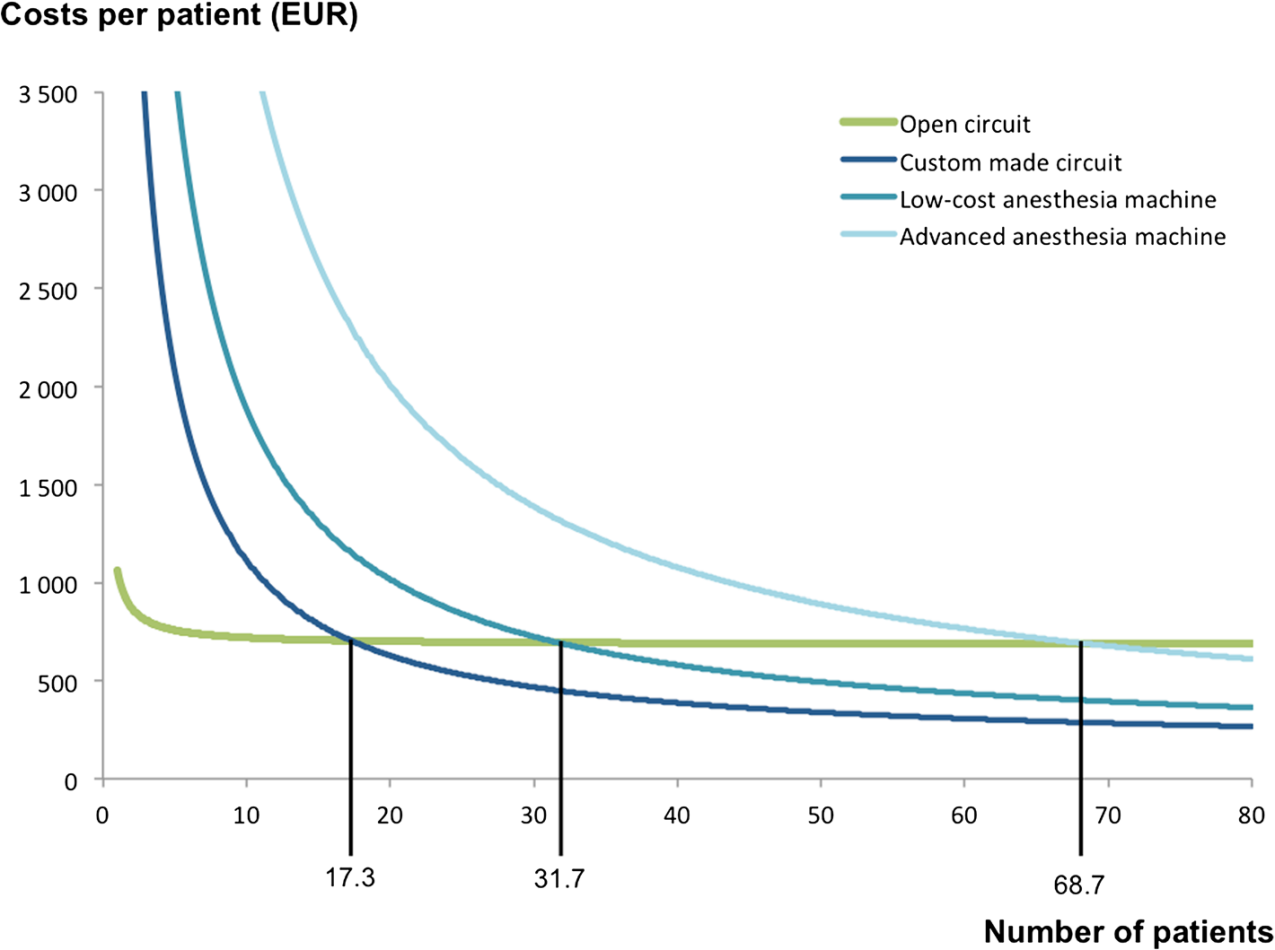
Dependency of heliox therapy cost per patient upon a number of patients treated

Figure 3 illustrates the total costs of heliox therapy with the four different heliox administration systems studied. The cost of a custom-made semi-closed circuit is recuperated after heliox application in 18 patients. Thereafter the therapy using the semi-closed circuit will be cheaper compared to the standard heliox therapy using an open circuit. The corresponding number of patients is 32 when a low-cost anesthesia machine is initially acquired and this number rises to 69 when a highly advanced anesthesia machine is considered.

**Fig. 4:**
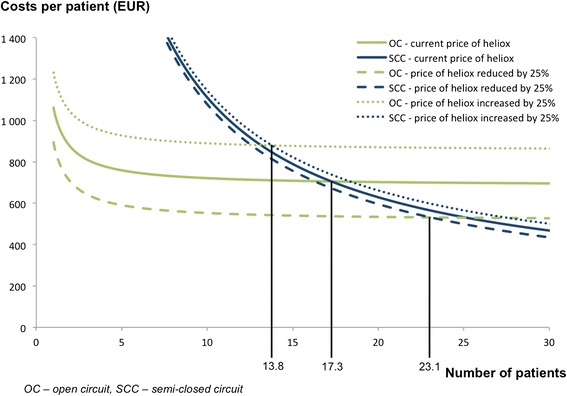
Results of sensitivity analysis considering changes in heliox price

Results of the sensitivity analysis presented in Fig. 4 show how the price of heliox affects the number of patients for which the initial cost of the custom-made semi-closed circuit is recuperated. This corresponds to ≥ 14 patients for the price of heliox to be increased by 25 %, and ≥ 24 patients for the price of heliox to be decreased by 25 %.

**Fig. 5:**
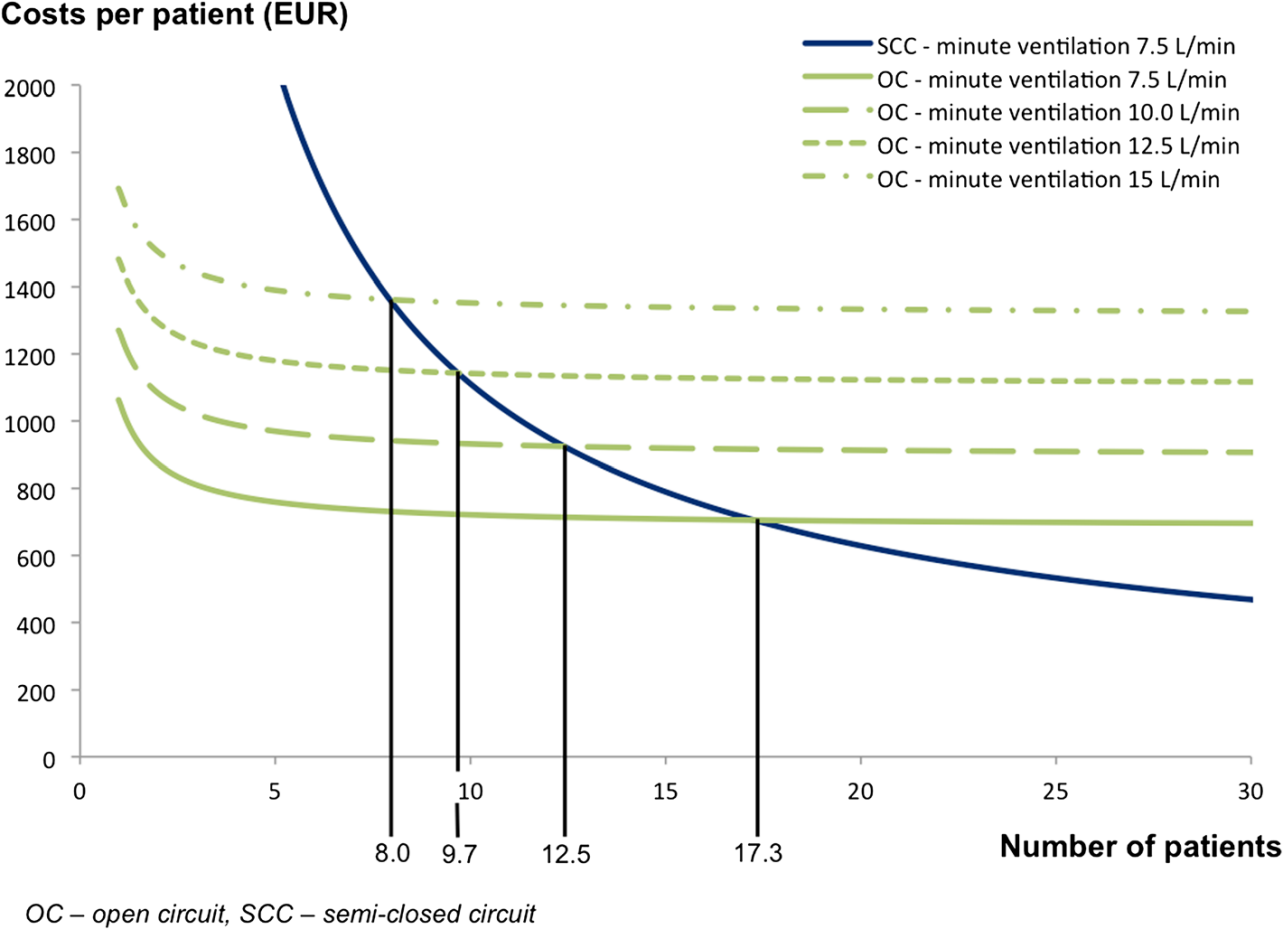
Results of sensitivity analysis considering changes in minute ventilation of the patients

An increase in minute ventilation of a patient over the average physiological value of 7.5 L/min is associated with higher fresh gas flow in an open circuit only, while CO_2_ absorber in a semi-closed circuit prevents CO_2_ rebreathing with any minute ventilation of a patient without affecting fresh gas flow rate into the circuit. Figure 5 compares the total costs of helium therapy per patient in the open circuit and in the custom made semi-closed circuit for various values of minute ventilation. The cost of the custom made semi-closed circuit is recuperated for 13, 10 and 8 patients when their minute ventilation increases to 10, 12.5 and 15 L/min respectively.

## Discussion

The main finding of this study is that a semi-closed circuit for heliox administration in spontaneously breathing patients reduces cost of heliox therapy significantly to one-fifth the cost compared to the standard way of heliox administration using an open circuit. If a new semi-closed circuit is needed, the initial cost of such a system is recuperated after heliox application in 18 patients when the system is assembled from standard components of anesthesia circuits. When a standard anesthesia machine is initially acquired, the cost is recuperated in 32–69 patients depending on the initial cost of the anesthesia machine.

The specified reduction in cost of the heliox therapy per patient using a semi-closed circuit is calculated while taking into account standard procedures, such as aerosol therapy, personal hygiene and nutrition, which disrupt the low-flow heliox therapy and thereby increase heliox consumption and cost.

As there are many possibilities how to acquire a suitable semi-closed system, three possibilities are considered in this study. The cheapest possibility is to assemble a semi-closed system from standard commercially available components of anesthesia machines. The calculated cost also includes the system assembly by a biomedical company. Use of a commercially available anesthesia machine represents a more expensive alternative. Since the modern anesthesia machines contain features that would not be utilized during the desired heliox therapy, the simplest system would be the most cost-effective option.

Airflow resistances of the inspiratory and expiratory limbs of a semi-closed circuit should be minimized as they increase patient’s imposed work of breathing, which represents a disadvantage of a semi-closed circuit. A substantial part of the imposed work of breathing may be caused by the high resistance of the expiratory valve (Adjustable Pressure Limiting valve). We reached the lowest resistance with a simple water seal, which is used for bubble continuous positive airway pressure [8]. Bacterial and viral breathing filters have relatively high airflow resistance that even increases when the filters become wet [9]. Therefore, the analyzed semi-closed systems did not contain any breathing filter. As a consequence, all components of the semi-closed circuits require sterilization after each patient. The costs of sterilization are therefore included in the cost analysis conducted in this study.

The custom-made semi-closed circuit was used in COPD patients and tested in a clinical trial approved by the State Institute for Drug Control, Czech Republic (EudraCT number: 2008-008274-31). For analysis conducted in this study we used published input data and our experience from the clinical trial. Since this study is a theoretical cost analysis, it does not describe the clinical trial and its evaluation.

The average length of heliox therapy was considered to be 25 h per patient in this study in concordance with the estimated time to onset of a steroid response to be as early as 2 to 4 h and as late as 1 to 2 days [10]. The duration was also determined based on our clinical experience with typical length of heliox therapy in COPD exacerbation patients.

Based on our previous clinical experience, the phase of denitrogenation should be approximately 5 min which was an average length of denitrogenation by a fresh gas flow rate of 8 L/min resulting in a concentration of helium higher then 70 % measured in the inspiratory limb of the semi-closed circuit. Even though the complete denitrogenation cannot be accomplished within 5 min, the traces of nitrogen washed out from the body are effectively eliminated from the semi-closed circuit during the subsequent ventilation with a relatively high heliox fresh gas flow rate of 3 L/min.

The basic economic calculations for the open circuit were conducted assuming standard condition that the fresh gas flow through an open circuit should be three times higher than the minute ventilation of a patient in order to avoid rebreathing of expired gas [11]. Considering the average minute ventilation in a healthy adult of 7.5 L/min [12], the basic fresh gas flow through the open circuit was assumed to be 22.5 L/min. In respiratory diseases such as COPD and bronchial asthma the minute ventilation increases significantly [13–15]. Therefore, the economic simulations for the open circuit were conducted for minute ventilations which were increased to 10 L/min, 12.5 L/min and 15 L/min. In contrast, an increase in minute ventilation of a patient does not affect the required fresh gas flow into the semi-closed circuit due to the rebreathing principle and exhaled CO_2_ removal.

During the low-flow heliox therapy, usage of a leak-free semi-closed circuit allows reduction of fresh gas flow to less then 1 L/min. Even though a high-quality sealed face mask is used, contamination of the semi-closed circuit by ambient air may decrease the helium concentration. A fresh gas flow of 3 L/min is high enough to compensate for the leak in the face mask. Based on our previous clinical experience, 3 L/min fresh gas flow assures that helium concentration in the semi-closed circuit is always higher than 70 % thus maximizing the positive helium effect in the obstructed airways.

Heliox 80:20 (80 % of helium and 20 % of oxygen mixture) was considered during the analysis. Oxygen fraction higher than 20 % reduces helium concentration and thereby has a negative effect on the airway resistance improvement caused by heliox. Although the lowest effective helium concentration is unknown, it is generally accepted to be more than 60 % to have positive physiological effects [16]. The system is designed for patients previously breathing room air spontaneously. We speculated that they will not require increased oxygen concentration because airway resistance reduction and improved gas exchange are expected immediately after heliox therapy initiation [17]. Therefore, we conducted all calculations using helium concentration of 80 %.

In some patients, multiple masks may be used in order to find a mask that properly fits and is comfortable for the patient. This fact increases costs of consumables and costs of sterilization. These extra costs have a negligible effect on the results of the study. Considering that 20 % of patients may require multiple masks, our cost analysis does not change.

The costs of electricity in all the fours assessed systems are very low (between 0.02 – 0.05 EUR/hour) and thus inconsequential. Therefore, we did not included it in our analysis.

The medical staff should be present in the ward throughout the whole heliox therapy regardless of the system used for heliox administration. The same is true even when an alternative therapy is employed. Therefore, the personal costs of the medical staff are identical in all the evaluated systems and were therefore not considered in this study.

The conducted sensitivity analysis showed that the semi-closed circuit will be more advantageous if there is an increase in price of heliox. On the other hand, if the price of heliox decreases, a semi-closed circuit will still be beneficial but will require additional patients to recuperate the initial cost. However, decrease in price of heliox is unexpected due to the increasing prices of medicinal gases and due to the fact the world’s helium resources are limited [18].

Usage of a semi-closed circuit decreases the consumption of heliox and thus reduces the cost of heliox therapy. This might boost a wider use of heliox in clinical practice and support clinical studies devoted to heliox applications in various clinical situations [19]. Despite physiologically favorable properties of heliox treatment for the patients with airway obstruction as compared to the air and oxygen treatment, there is still not sufficient evidence that it is suitable for the large scale use. Therefore, the impact of finding a theoretic cheaper way of heliox administration on the clinical practice needs to be ascertained.

## Conclusions

Heliox therapy in spontaneously breathing patients using a semi-closed circuit becomes more cost-effective compared to the open circuit, currently used in clinical practice, when applied in a sufficient number of cases. This effect is enhanced with increasing number of patients treated.

## References

[1] Tobin MJ (2012). Principles and Practice of Mechanical Ventilation.

[2] Hess D (2006). Heliox and noninvasive positive-pres sure ventilation: A role for heliox in exacerbations of chronic obstructive pulmonary disease?. Respir Care.

[3] Frazier MD, Cheifetz IM (2010). The role of heliox in paediatric respiratory disease. Paediatr Respir Rev.

[4] Eves ND, Ford GT (2007). Helium–oxygen: A versatile therapy to “lighten the load” of chronic obstructive pulmonary disease (COPD). Respir Med COPD Updat.

[5] Roche-Campo F, Vignaux L, Galia F, Lyazidi A, Vargas F, Texereau J (2011). Delivery of helium–oxygen mixture during spontaneous breathing: evaluation of three high-concentration face masks. Intensive Care Med.

[6] Fleming M, Weigelt J, Brewer V, McIntire D (1992). Effect of Helium and Oxygen on air-flow in a narrowed airway. Arch Surg.

[7] Roubik K, Zazula R, Strnadova A (2012). Spontaneous breathing of heliox using a semi-closed circuit: A bench study. Int J Artif Organs.

[8] Urs PS, Khan F, Maiya PP (2009). Bubble CPAP - A Primary Respiratory Support for Respiratory Distress Syndrome in Newborns. Indian Pediatr.

[9] Turnbull D, Fisher PC, Mills GH, Morgan-Hughes NJ (2005). Performance of breathing filters under wet conditions: a laboratory evaluation. Br J Anaesth.

[10] Bullard MJ, Liaw SJ, Tsai YH, Min HP (1996). Early corticosteroid use in acute exacerbations of chronic airflow obstruction. Am J Emerg Med.

[11] Kaul TK, Mittal G (2013). Mapleson’s Breathing Systems. Indian J Anaesth.

[12] West JB: Best and Taylor’s Physiological Basis of Medical Practice. 12th edition. Baltimore: Williams & Wilkins; 1990:1170.

[13] Piquilloud L, Tassaux D, Bialais E, Lambermont B, Sottiaux T, Roeseler J (2012). Neurally adjusted ventilatory assist (NAVA) improves patient-ventilator interaction during non-invasive ventilation delivered by face mask. Intensive Care Med.

[14] Ballester E, Reyes A, Roca J, Guitart R, Wagner PD, Rodriguez-Roisin R (1989). Ventilation-perfusion mismatching in acute severe asthma: effects of salbutamol and 100 % oxygen. Thorax.

[15] Boeck L, Tamm M, Grendelmeier P, Stolz D (2012). Acute effects of aerosolized iloprost in COPD related pulmonary hypertension - a randomized controlled crossover trial. PLoS One.

[16] Chatmongkolchart S, Kacmarek RM, Hess DR (2001). Heliox delivery with noninvasive positive pressure ventilation: a laboratory study. Respir Care.

[17] Gentile MA (2011). Inhaled medical gases: more to breathe than oxygen. Respir Care.

[18] Adee S (2010). Physics Projects Deflate for Lack of Helium-3 [Update. IEEE Spectr.

[19] Gainnier M, Forel J-M (2006). Clinical review: use of helium-oxygen in critically ill patients. Crit Care.

